# Impact of antral follicle count on follicular–luteal characteristics, superovulatory response, and embryo quality in Sahiwal cows

**DOI:** 10.3389/fvets.2024.1494065

**Published:** 2024-10-22

**Authors:** Mohan Gawai, Brijesh Kumar, S. Mehrotra, Pradeep Chandra, Kalpendra Kohli, Manoj Donadkar, Vandana Yadav, Brijesh Kumar Yadav, Chinmay Warghat, Nitish Kharayat, Dushyant Yadav, Sumit Singhal, V.S. Chouhan, S.K. Singh, M.H. Khan

**Affiliations:** ^1^Animal Reproduction Division, ICAR-Indian Veterinary Research Institute (ICAR-IVRI), Bareilly, India; ^2^Livestock Production and Management Section, ICAR-IVRI, Bareilly, India; ^3^Department of Teaching Veterinary Clinical Complex, DUVASU, Mathura, India; ^4^Temperate Animal Husbandry Division, ICAR-IVRI, Mukteswar Campus, Bareilly, India; ^5^Department of Veterinary Gynaecology and Obstetrics, BASU, Patna, India; ^6^Department of Veterinary Physiology and Climatology, ICAR- IVRI, Bareilly, India

**Keywords:** antral follicle counts, superovulatory follicular development, embryo quality, Sahiwal cattle, luteal development

## Abstract

The study aimed to evaluate the effect of antral follicle count (AFC) on follicular and luteal development during the estrous cycle and superovulatory period, as well as on superovulatory response and *in vivo* embryo quality within the MOET program. A total of 48 estrus-induced (500 μg PGF2α, Single dose, IM) Sahiwal cows (*Bos indicus*) with a BCS between 3.5 and 4.0 were selected for the study. On the day of wave emergence, the animals were divided into two groups based on the AFC, i.e., low AFC (≤18) and high AFC (>18). Both the groups were monitored daily using B-mode ultrasonography (USG) for one cycle, and the superovulation protocol was initiated on the 9th day of the subsequent estrous cycle. A total of 240 μg of FSH in eight divided doses were given in a tapering sequence for 4 days and simultaneous administration of 500 μg PGF2α, along with the fifth dose of FSH. Donors were inseminated at superovulatory estrus using double straws of high-quality frozen semen thrice at 12-h intervals, and non-surgical flushing was performed on day 7 of the superovulatory estrus followed by embryo searching and evaluation under a stereo zoom microscope. Ovulatory waves of the high-AFC Sahiwal cows have significantly (*p* ≤ 0.05) larger sizes of preovulatory follicles (POF) (12.06 ± 0.19 mm *vs* 11.56 ± 0.16 mm) and corpus luteum (CL) (19.57 ± 0.28 mm *vs* 18.26 ± 0.35 mm), as compared to low AFC. The ovarian size was significantly (*p* < 0.0001) larger in cows with high AFC during the superovulatory protocol. The number of large, medium, and small follicles was significantly (*p* < 0.0001) high on the day of superovulatory estrus (SOE), PGF2α administration, and initiation of superovulatory protocol, respectively, in high AFC. Donors with high AFC had a notably greater (*p* < 0.0001) count of CL and embryos retrieved per flushing, including excellent and fair-quality embryos. A strong association (*p* < 0.0001) between high AFC and ovarian size (*r* = 0.9136), superovulatory response (*r* = 0.9350), and embryo quality (x^2^ = 8.788; *p* = 0.032) and number (*r* = 0.9858) were also recorded. Based on these results, AFC is considered a dependable indicator for forecasting reproductive capacity. *Bos indicus* donors with an average AFC of 30 or higher are recommended.

## 1 Introduction

Reproductive biotechnologies, such as embryo production, play a crucial role in enhancing the reproductive efficiency of cattle over a short period. While the utilization of *in vitro* embryo production (IVEP) has grown significantly over the past decade, embryo production through multiple ovulation and embryo transfer (MOET—*in vivo* method) programs remains a substantial portion of global cattle embryo production (1). To harvest the maximum potential of assisted reproductive techniques (ART), it is crucial to have dependable biomarkers that demonstrate strong consistency and heritability and are linked to the reproductive performance of cows (2, 3). Intrinsic and extrinsic factors govern the success of a MOET program, including season, genetics, age, nutrition, management, stress, type of gonadotropins used, and treatment protocols adopted (4). Despite improved control over external factors, the inconsistency in the ovarian response indicates that intrinsic factors are primarily responsible for this variability (5).

In this context, the antral follicle count (AFC), which reflects the population of antral follicles present in an ovary, has been indicated as an important phenotypic characteristic related to female fertility and positively correlated with the superovulatory response and performance of *in vivo* and *in vitro* embryo production (IVEP) (6, 7). Over time, there has been a growing understanding of pharmacological strategies to manage the estrous cycle, the physiological processes of ovarian superstimulation using hormonal protocols (8), and various factors influencing embryo production (9), allowing a choice of strategies and the early control of factors that can improve embryo yield [Bo (10)]. Among the various factors influencing the *in vivo* production of bovine embryos (9, 10), the antral follicle count (AFC) stands out as one of the most significant and is closely related to donor selection (7). Once a female’s high genetic potential is confirmed (11), AFC can serve as a criterion for selecting donors with a high AFC (6, 12). A significant correlation (r = 0.88) has been discovered between anti-Müllerian hormone (AMH) and AFC, with both being regarded as markers of ovarian response (13–15).

AFC and serum AMH levels are reproductive parameters that exhibit significant variability among females but demonstrate high consistency within the same animal (16, 17). Additionally, while AMH measurement requires laboratory analysis, the AFC can be assessed via a single ultrasound examination of both ovaries by a trained operator at any time in the cycle to classify females based on the AFC numbers (16). *Bos indicus* and *Bos taurus* cattle appear to have different fertility responses based on their AFC category. Moreover, females with high AFC showed a greater number of embryos produced by the donors in *Bos taurus* (18), crossbred *indicus-taurus* (17), and *Bos indicus* (19). Additionally, cows with high AFC had higher conception rates than those with low AFC (20, 21).

Considering the application of AFC as a valuable tool to assist cattle performance and reproductive biotechnology, thus the aim of this study was to determine follicular and luteal development in relation to AFC and its association with ovarian size, superovulatory response, and embryo quality and number in Sahiwal donor (*Bos indicus*) subjected to a MOET program.

## 2 Materials and methods

### 2.1 Location, animals, and management

A total of 48 Sahiwal cows (*Bos indicus*) of 1–4 parity, minimum 60 days postpartum, with a body condition score (BCS) between 3.5 and 4.0 on a scale of 1–5 (22) with a mean live weight 345 ± 6 kg (minimum 290, maximum 410 kg) acted as embryo donor. These cows were maintained at Cattle and Buffalo Farm, ICAR-IVRI, Izatnagar, located at an altitude of 564 meters above mean sea level, at latitude and longitude of 28° N and 79°E, respectively.

The total experiment was conducted during the winter months (November to March). All cows were fed 3.5–4.5 kg of concentrate feed per day, containing 20% digestible crude protein (DCP) and 70% total digestible nutrients (TDN), had *ad libitum* access to fresh drinking water, and wheat straw, and were provided green fodder twice a day. All the animals were maintained under hygienic and optimal management conditions in a semi-intensive system with access to a large, open paddock for free movement. Heat detection in the herd was carried out twice daily at 07:00 and 16:00 using a vasectomized bull (teaser) accompanied by skilled herdsmen. Health and vaccination protocols were followed as per the standard schedule of farm management.

### 2.2 Experimental design

A total of 48 estrus-induced (500 μg PGF2α, Estrumate™, MSD animal health, India, Single dose, IM) Sahiwal cows (*Bos indicus*) with a BCS between 3.5 and 4.0 were selected for the study from the herd. Both the right and left ovaries (the pair) were scanned ultrasonographically with a 7.5-MHz transducer (Exago ECM, France), and on the day of wave emergence, antral follicles (all follicles >2 mm) were counted to determine the total number of antral follicles as previously described by Morotti et al. (16).

Donors were categorized into two groups based on their AFC results. 48 donors were chosen from the herd, with 24 having a consistently low AFC (≤ 18 follicles; mean = 13.21 ± 0.90 follicles; range = 8–18 follicles) and the other 24 having a consistently high AFC (> 18 follicles; mean = 30.08 ± 1.64 follicles; range = 19–46 follicles). Both groups were monitored daily using a B-mode ultrasonography (USG) for one cycle to record follicular and luteal development. The superovulation protocol was initiated on the ninth day of the following estrous cycle.

**Figure 1:**
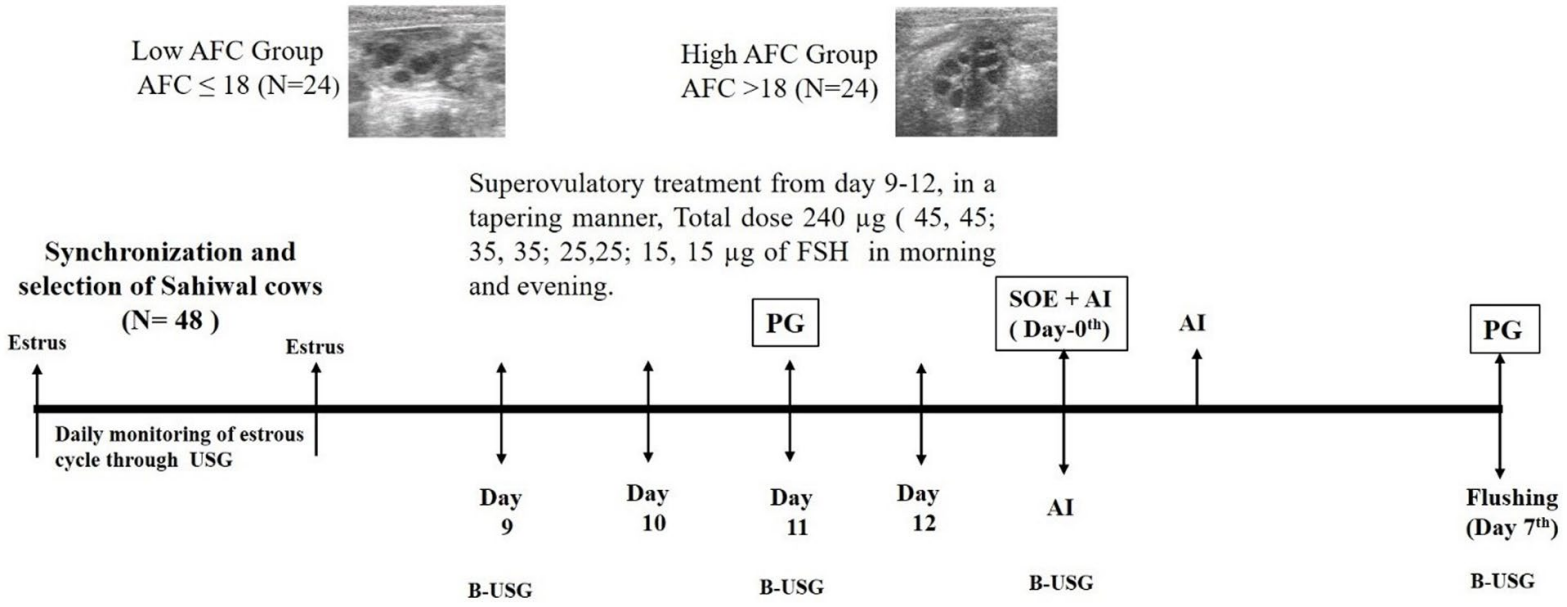
Experimental design, B-mode ultrasound monitoring of follicular and luteal development in high- and low-AFC Sahiwal cows (*Bos indicus*). Evaluations were performed at the same time, and ovulation was determined by the disappearance of POF in the ultrasound images. Superovulatory protocol was started on day 9 of the subsequent estrus. Non-surgical flushing was performed on day 7 post-superovulatory AI. AFC, Antral follicle count; USG, ultrasonography; PG, prostaglandin F2 alpha; SOE, superovulatory estrus; AI, artificial insemination.

Intramuscular injections of STIMUFOL^®^ (Reprobiol SPRL, Belgium), of a total dose of 240 μg in eight fragmented doses (45,45; 35,35; 25,25; 15,15 μg morning and evening) were given in tapering sequence for 4 days and simultaneous administration of 500 μg PGF2α, along with the 5^th^ dose of FSH. Donors were inseminated during superovulatory estrus using double straws of high-quality frozen semen, thrice at 12 h. intervals. Non-surgical flushing of both horns was carried out on the 7th day of the superovulatory estrus (Figure 1) using a Woerlein catheter (IMV Technology, India), followed by embryo searching and evaluation under a stereo zoom microscope (SMZ 1000, Nikon, Japan). The embryo quality has been determined in accordance with the International Embryo Technology Society (IETS) standards.

### 2.3 Statistical analysis

All data from the high- and low-AFC groups were analyzed for mean and standard error (SE) using the GraphPad Prism 8.0.1 software module. The different follicular and luteal developmental attributes in both groups were analyzed using an unpaired *t*-test. The significance among different groups or days was assessed through *post-hoc* Tukey’s testing. For descriptive analysis, the data were presented as the mean and standard (M ± SE). Pearson’s correlation analysis was applied to explore the potential relationship of AFC with ovarian size, superovulatory response, and embryo production. The significance level for rejecting H0 (the null hypothesis) was 5%; therefore, a significance level of *p* ≤ 0.05 was considered to indicate an effect of the categorical variables and their interaction, whereas a significance level of *p* > 0.05 indicated a lack of statistical significance.

## 3 Results

### 3.1 Follicular characteristics in non-ovulatory and ovulatory waves

The wave emergence in high-AFC Sahiwal cows was significantly (*p* = 0.04) earlier however, deviating late (*p* = 0.03) with a larger size of the dominant follicle in the non-ovulatory wave (Table 1). The follicular attributes of subordinate follicles of non-ovulatory waves of high- and low-AFC Sahiwal cattle did not vary significantly (*p* > 0.05) (Table 2). Similarly, ovulatory waves of high-AFC Sahiwal cows have significantly (*p* = 0.04) larger sizes of preovulatory follicles as compared to low-AFC Sahiwal cows (Table 3). Furthermore, the inter estrus period, inter ovulatory interval (Table 3), and various attributes of the subordinate follicle of ovulatory wave did not significantly differ (*p* > 0.05) between high- and low-AFC Sahiwal cows (Table 4).

**Table 1:** Follicular characteristics (Mean ± SEM) of non-ovulatory wave of high- and low-AFC Sahiwal cows.

Follicular characteristics	High AFC (*N* = 24)	Low AFC (*N* = 24)	*p-*value
Day of wave emergence	0.13 ± 0.15	0.67 ± 0.21	0.04
No. of follicles at wave emergence	29.5 ± 1.55	14.25 ± 0.73	0.00
Day of deviation	4.0 ± 0.30	3.29 ± 0.19	0.04
Diameter at deviation (mm)	8.39 ± 0.21	8.02 ± 0.18	0.18
Dominant follicle maximum diameter (mm)	11.95 ± 0.24	10.83 ± 0.16	0.03
Day of maximum diameter	6.83 ± 0.37	7.38 ± 0.42	0.34
Growth rate (mm/day)	1.07 ± 0.07	0.98 ± 0.03	0.28
Growth period (days)	5.54 ± 0.35	6.25 ± 0.33	0.15
Onset of regression (day)	8.00 ± 0.36	8.54 ± 0.38	0.31
Regression rate (mm/day)	0.89 ± 0.05	0.91 ± 0.06	0.77
Regression period (days)	6.21 ± 0.32	6.17 ± 0.42	0.94
Duration of wave (days)	12.50 ± 0.60	13.92 ± 0.60	0.10

N, Number of Sahiwal cows; AFC, antral follicle count; SEM, standard error of the mean.

**Table 2:** Follicular characteristics (mean ± SEM) subordinate follicles of non-ovulatory wave of high- and low-AFC Sahiwal cows.

Follicular characteristics	High AFC (*N* = 24)	Low AFC (*N* = 24)	*P-*value
Subordinate follicle maximum diameter (mm)	7.16 ± 0.13	7.16 ± 0.16	0.97
Growth rate (mm/day)	0.78 ± 0.05	0.73 ± 0.03	0.51
Growth period (days)	3.00 ± 0.16	3.38 ± 0.25	0.21
Regression rate (mm/day)	0.83 ± 0.05	0.81 ± 0.04	0.81
Regression period (days)	2.88 ± 0.16	3.17 ± 0.12	0.15

N, Number of Sahiwal cows; AFC, antral follicle count; SEM, standard error of the mean.

**Table 3:** Follicular characteristics (mean ± SEM) of ovulatory wave of high- and low-AFC Sahiwal cows.

Follicular characteristics	High AFC (*N* = 24)	Low AFC (*N* = 24)	*P*-value
Day of wave emergence	10.96 ± 0.33	10.25 ± 0.31	0.13
No. of follicles at wave emergence	30.08 ± 1.64	13.21 ± 0.90	**<**0.0001
Day of deviation	14.75 ± 0.41	13.88 ± 0.32	0.10
Diameter at deviation (mm)	7.98 ± 0.20	8.03 ± 0.14	0.84
Growth rate (mm/day)	1.09 ± 0.06	0.95 ± 0.48	0.11
Duration of wave (days)	11.25 ± 0.49	11.92 ± 0.52	0.36
Size of preovulatory follicle (mm)	12.06 ± 0.19	11.56 ± 0.16	0.04
Day of maximum diameter of POF	20.21 ± 0.38	19.79 ± 0.59	0.56
Inter estrus period (days)	20.33 ± 0.21	19.71 ± 0.35	0.13
Inter-ovulatory interval (days)	21.38 ± 0.27	20.67 ± 0.40	0.15

N, Number of Sahiwal cows; AFC, antral follicle count; SEM, standard error of the mean.

**Table 4:** Follicular characteristics (mean ± SEM) subordinate follicles of ovulatory wave of high- and low-AFC Sahiwal cows.

Follicular characteristics	High AFC (*N* = 24)	Low AFC (*N* = 24)	*P-*value
Subordinate follicle maximum diameter (mm)	7.29 ± 0.10	7.1 ± 0.14	0.27
Growth rate (mm/day)	0.97 ± 0.06	0.81 ± 0.06	0.34
Growth period (days)	3.20 ± 0.22	3.29 ± 0.22	0.90
Regression rate (mm/day)	0.74 ± 0.04	0.78 ± 0.03	0.40
Regression period (days)	2.67 ± 0.13	2.58 ± 0.15	0.67

N, Number of Sahiwal cows; AFC, antral follicle count; SEM, standard error of the mean.

### 3.2 Luteal characteristics of high- and low-AFC donors

The day of CL detection was significantly (*p* = 0.03) late with a larger (*p* = 0.001) diameter at detection as well as the maximum diameter (*p* = 0.01) of CL was reported in the high-AFC group (Table 5).

**Table 5:** Luteal characteristics (Mean ± SEM) during estrous cycle of high- and low-AFC Sahiwal cows.

Luteal characteristics	High AFC (*N* = 24)	Low AFC (*N* = 24)	*P-*value
Day of detection of CL	3.13 ± 0.20	2.42 ± 0.24	0.03
Diameter at detection (mm)	10.04 ± 0.37	9.12 ± 0.19	0.001
Day of maximum diameter of CL	12.42 ± 0.44	12.13 ± 0.68	0.72
Maximum diameter of CL (mm)	19.57 ± 0.28	18.26 ± 0.35	0.01
CL growth rate (mm/day)	1.09 ± 0.09	0.97 ± 0.08	0.34
Growth period (day)	9.33 ± 0.46	10.58 ± 0.52	0.08
Regression rate (mm/day)	0.93 ± 0.04	1.38 ± 0.07	<0.0001
Regression period (day)	6.50 ± 0.41	5.75 ± 0.30	0.15
Lifespan of CL (day)	18.00 ± 0.39	18.71 ± 0.50	0.27

N, Number of Sahiwal cows; AFC, antral follicle count; SEM, standard error of the mean.

### 3.3 Ovarian size of high- and low-AFC donors

The ovarian size (diameter) of high-AFC Sahiwal donors showed significantly (*p* < 0.0001) larger measurements than low AFC on the day of estrus, during the superovulatory protocol, and on the day of flushing (Table 6).

**Table 6:** Effect of AFC on ovarian size (mm) during superovulatory protocol (mean ± SE) in Sahiwal cows.

Different days of super ovulatory protocol	High AFC (N = 24)	Low AFC (N = 24)	*P-*value
D-estrus	22.49 ± 0.32	20.78 ± 0.20	<0.0001
D1-FSH	23.42 ± 0.27	21.12 ± 0.24	<0.0001
D-PG	26.81 ± 0.41	24.27 ± 0.35	<0.0001
D-SOE	33.33 ± 0.37	27.81 ± 0.34	<0.0001
D-flushing	41.95 ± 0.75	32.61 ± 0.58	<0.0001

D-estrus, Day of estrus; D1-FSH, day of initiation of superovulatory treatment; D-PG, day of prostaglandin injection; D-SOE, day of superovulatory estrus; N, number of Sahiwal cows; AFC, antral follicle count; SEM, standard error of the mean.

### 3.4 Follicular development during the superovulatory period

Large, medium, and small follicles were significantly (*p* < 0.0001) higher on the day of superovulatory estrus (SOE), the day of PGF2α injection, and the day of initiation of superovulatory treatment, respectively, in high-AFC cattle than low-AFC cattle (Table 7). The low-AFC cattle attained significantly (*p* = 0.03) larger POF size at SOE than high AFC, and no significant (*p* > 0.05) differences were observed in medium-size follicles between the groups (Table 8).

**Table 7:** Various types of follicles (mean ± SEM) at different days of superovulatory protocol in high- and low-AFC Sahiwal cows.

Different days of superovulatory protocol	High AFC (*N* = 24)	Low AFC (*N* = 24)	*P-*value
Large-size follicle			
D1-FSH	0.71 ± 0.09	0.67 ± 0.13	0.80
D-PG	2.46 ± 0.26	1.71 ± 0.16	0.02
D-SOE	15.83 ± 1.04	8.92 ± 0.68	<0.0001
Medium-size follicle			
D1-FSH	2.29 ± 0.37	1.29 ± 0.11	0.02
D-PG	17.25 ± 1.17	9.08 ± 0.57	<0.0001
D-SOE	3.21 ± 0.23	2.79 ± 0.17	0.16
Small-size follicle			
D1-FSH	24.42 ± 1.32	12.75 ± 0.63	<0.0001
D-PG	3.46 ± 0.52	3.12 ± 0.22	0.56

D1-FSH, Day of initiation of superovulatory treatment; D-PG, day of prostaglandin injection; D-SOE, day of superovulatory estrus; N, number of Sahiwal cows; AFC, antral follicle count; SEM, standard error of the mean.

**Table 8:** Size of various types of follicles (mean ± SEM) at different days of superovulatory protocol in high- and low-AFC Sahiwal cows.

Different days of superovulatory protocol	High AFC (*N* = 24)	Low AFC (*N* = 24)	*P-*value
Large-size follicle (mm)			
D1-FSH	9.07 ± 0.93(n = 20)	9.21 ± 0.97(n = 19)	0.35
D-PG	9.48 ± 0.43	9.72 ± 0.16	0.61
D-SOE	10.34 ± 0.10	10.70 ± 0.12	0.03
Medium-size follicle (mm)			
D1-FSH	6.63 ± 0.53	7.07 ± 0.33	0.51
D-PG	7.34 ± 0.09	7.25 ± 0.09	0.53
D-SOE	7.56 ± 0.18	7.37 ± 0.45	0.47

D1-FSH, Day of initiation of superovulatory treatment; D-PG, day of prostaglandin injection; D-SOE, day of superovulatory estrus; N, number of Sahiwal cows; AFC, antral follicle count; SEM, standard error of the mean.

### 3.5 Superovulatory response

The total number of CL was significantly (*p* < 0.0001) higher in high-AFC cattle; however, there was no significant (*p* > 0.05) difference in the diameter of CL between the groups on the day of flushing (Table 9).

**Table 9:** Effect of AFC on superovulatory response (mean ± SEM) in Sahiwal cows.

Parameters (on the day of flushing)	High AFC (*N* = 24)	Low AFC (*N* = 24)	*P-*value
Number of CL	11.25 ± 0.69	6.04 ± 0.46	<0.0001
Mean diameter of CL (mm)	12.88 ± 0.25	12.24 ± 0.25	0.08

N, Number of Sahiwal cows; AFC, antral follicle count; SEM, standard error of the mean; CL, corpus luteum.

### 3.6 Embryo production and quality

The number of embryos recovered per flushing with excellent and fair-quality embryos (Transferable) was significantly (*p* ≤ 0.05) higher in high-AFC Sahiwal donors (Tables 10, 11). AFC was positively correlated to the ovarian size (*r* = 0.9136 *p* < 0.0001), superovulatory response (*r* = 0.9350 *p* < 0.0001), and embryo production (*r* = 0.9858 *p* < 0.0001) (Table 12).

**Table 10:** Effect of AFC on embryo recovered per flushing and its quality (mean ± SE) in Sahiwal cows.

Parameters	High AFC (N = 15)	Low AFC (*N* = 17)	*P-*value
Embryo recovered per flushing	7.46 ± 1.12	3.88 ± 0.42	0.0039
Embryo quality			
Excellent or good	4.4 ± 0.67	2.29 ± 0.50	0.0161
Fair	1.6 ± 0.34	0.71 ± 0.21	0.0191
Poor	0.66 ± 0.12	0.81 ± 0.18	0.529
Dead and degenerated	0.33 ± 0.12	0.35 ± 0.11	0.9109

N, Number of Sahiwal cows flushed; AFC, antral follicle count; SEM, standard error of the mean.

**Table 11:** Effect of AFC on total recovered embryo and its quality in Sahiwal cows.

Parameters	High AFC (*N* = 15)	Low AFC (*N* = 17)	*P-*value
Total no. of embryos recovered	112	66	X^2(df,1)^ = 8.788^a^
			*P* = 0.032 Likelihood ratio = 0.036 Linear-by-Linear Association = 0.005
Embryo quality			
Excellent or good	63.39% (71/112)	48.4% (32/66)	
Fair	25% (28/112)	22.7% (15/66)	
Poor	8.9% (10/112)	19.6% (13/66)	
Dead and degenerated	4.4% (3/112)	9.09% (6/66)	

N, Number of Sahiwal cows flushed; AFC, antral follicle count; SEM, standard error of the mean.

**Table 12:** Pearson’s correlation (r) for the association of AFC with ovarian size, superovulatory response, and embryo production and the significance score for each variable.

Variable	Ovarian size		Superovulatory response		Embryo production	
	*r*	*p*	*r*	*p*	*r*	*p*
High AFC	0.9136	<0.0001	0.9350	<0.0001	0.9858	<0.0001
Low AFC	0.9011	<0.0001	0.9304	<0.0001	0.9589	<0.0001

## 4 Discussion

The present study reveals cows with high AFC showed larger POF, and follicles with large diameters before ovulation are associated with better reproductive performance in bovine females (23). According to USG studies, a high AFC may be related to superior reproductive performance, such as increased fertility, shorter open period, and higher reactivity to superovulation treatment in cows (21, 24, 25). In this regard, an increase in the number of 3–8-mm-sized antral follicles is expected to reflect the size of the ovaries. High AFC is related to several characteristics such as larger ovaries with higher possibilities of pregnancy toward the end of breeding seasons (21).

The number of follicles at wave emergence in both non-ovulatory and ovulatory waves of high-AFC cattle was comparable to the findings of Morotti et al. (11) and de Lima et al. (26) in Nelore cows and higher than in Holstein cows (27, 28). Furthermore, Ireland et al. (24) also recorded similar results in beef heifers and stated that the number of total follicles during the non-ovulatory and ovulatory waves were 2-fold higher in the high-AFC group than in the low-AFC group. A high degree of variability in AFC is evident and can be attributed to multiple factors such as the day of the estrous cycle (18), age (29), and the body condition of the animal (30, 31), genetics (32), maternal environment, and health (13, 20, 33). In the present study, cattle with high AFC exhibited a larger size of the dominant follicle, which is contradictory to the findings of Santos et al. (19) in Indicus-taurus cows; Morotti et al. (11) and de Lima et al. (26) in Nelore cows; and Bonato et al. (27) in Holstein cows noticed that cows with low AFC exhibited larger preovulatory follicles than high-AFC cows. Larger POF diameter in low-AFC cattle has been attributed to fewer follicles receiving gonadotropin stimulation, it is possible to expect that each follicle would obtain a larger amount of FSH at the emergence of the follicular wave. Conversely, those females with a high AFC would have more follicles to share the same amount of gonadotropins (14, 26). Larger size preovulatory follicles in the high-AFC group of the present study might be due to the large number of granulosa cells and higher concentration of estradiol (28). Scheetz et al. (34) reported that the cultured granulosa cells derived from the low-AFC cows showed lower secretion of estradiol, which is essential for granulosa cell proliferation. Angiogenic factors, such as endothelial nitric oxide synthase (eNOS), are expressed in ovarian follicles and involved in folliculogenesis, steroidogenesis, oocyte maturation, ovulation, and ultimately embryo development through the production of a free radical gas, nitric oxide (NO) (35–38). No significant difference in the diameter of subordinate follicles between the groups is in agreement with Burns et al. (29) and Ireland et al. (24) in beef heifers. In the present study, significant differences in the day and diameter of CL at detection with early detection in the low-AFC group were recorded. The maximum diameter of CL differed significantly between the groups with greater diameter in the high-AFC group that exhibited a larger diameter of POF. Similar findings were reported by Vasconcelos et al. (39) and Baruselli et al. (40) that ovulation of smaller size preovulatory follicles results in smaller CL that secrete less progesterone compared to larger CL. The difference in the diameter of CL may be due to the difference in the size of the preovulatory follicle. In contrast to the present study, Bonato et al. (27) in Holstein cows and Lima et al. (26) in *Bos indicus* found that cows with low AFC displayed larger preovulatory follicles and CL in comparison with the high-AFC group. The larger diameter and relatively faster growth rate of CL in the high-AFC group are probably due to the high vascularity of granulosa cells with increased angiogenic and growth factors in developing CL.

The number of medium and large follicles on the day of PGF2α injection and SOE were significantly high in the high-AFC group as compared to the low-AFC group, respectively. Corresponding to the present findings, Singh et al. (41) reported a positive correlation between the numbers of follicles measuring 5–7 mm and > 8 mm or larger at the end of the ovarian superstimulation process in the high-AFC crossbred Hereford cows than the low AFC. The larger size of medium and large follicles on the day of SOE corresponds to the findings of Tessaro et al. (42) reported that the greater size of large and medium follicles may be attributed to increased blood flow rates in developing follicles of high-AFC animals.

We recorded a significant difference in the total number of CL on the day of flushing and the mean value was greater in the high-AFC group than the low-AFC group, which aligned with the results of Ireland et al. (24) who documented that following ovarian stimulation the taurus donors with a high AFC had a greater number of CL than those with a low AFC on the day of flushing. Similar results were reported in Sahiwal (43) and in Nelore donors (7). Center et al. (44) recorded that the high-AFC cows expressed greater superovulatory response and produced a higher number of embryos as compared to low-AFC cows.

Ovarian follicular population or reserve is directly associated with superovulatory response. The AFC at the time of the start of FSH administration had a positive and significant correlation with the superovulatory response, indicating that cows having higher AFC at the start of FSH, developed more CL (18, 19). The growth of small antral follicles and their maturation and ovulation is supported by exogenous FSH and PMSG causing multiple ovulations therefore the population of small antral follicles at the beginning of stimulation is positively correlated with superovulatory response (19).

The ovarian size (diameter) of high-AFC Sahiwal donors showed significantly larger measurements than low AFC on the day of estrus, during the superovulatory protocol, and on the day of flushing. This is in accordance with the findings of Ireland et al. (18), who reported that animals with a consistently low AFC had significantly smaller wet weights of ovaries as well as smaller ovarian heights and lengths than those with a high AFC during follicular waves in Hereford x Angus x Charolais crossbred beef heifers. Morotti et al. (16) observed similar results in *Bos indicus* cattle, finding that cows with a high AFC had a larger diameter and ovarian area than those with a low AFC. Martinez et al. (45) found a similar correlation between AFC and ovarian area in *Bos taurus* cattle.

The high-AFC group yielded a greater number of total embryos, embryos recovered per flushing, excellent, and fair-quality (transferable) embryos, and a smaller proportion of poor and dead or degenerated embryos as compared to low-AFC Sahiwal donors. This is in agreement with a previous study by Morotti et al. (14). They pointed out that the early embryos retrieved on day 7 were greater in number and their quality was significantly better in donors belonging to the high-AFC group than those in the low-AFC group.

A positive and significant correlation of AFC with ovarian size, superovulatory response, and total embryo production with a higher number of transferable embryos was recorded in the Sahiwal donor of the present study. Morotti et al. (46) observed comparable results in Indicus-Taurus animals, where they found that donors with high AFC had a notably greater number of embryos per collection (6.9 ± 5.3) in contrast to those with lower AFC (1.9 ± 2.1). Silva-Santos et al. (17) also reported comparable results in Braford donors. On the other hand, Lollato et al. (7) reported contradictory findings regarding embryo production and quality. They noted that Nelore donors with either low or high AFC showed comparable outcomes regarding the total counts of viable and freezable embryos, along with the incidence of degenerated embryos.

A positive but non-significant result between the AFC group and the total number of recovered and transferable embryos was reported by Imtiyaz et al. (43). Similar findings have been noted in *in vitro* embryo production (IVEP), where donors with a high AFC demonstrated significantly higher production of oocytes and embryos via ovum pick up (OPU) in both *Bos taurus* and *Bos indicus* cattle (12, 17, 19, 47). This observation could be explained by the higher proportion of mature oocytes in the high-AFC group, which exhibited increased mitochondrial activity prior to maturation. This led to enhanced ova viability and higher rates of cleavage and blastocyst development (6, 48). Another possible reason for the superior embryo quality in high-AFC donors could be linked to changes in lipid composition. Rosa et al. (49) found that embryos retrieved from high-AFC Nelore donors exhibited increased triglyceride levels and decreased concentrations of cholesterol and diacylglycerol compared to embryos from low-AFC cows. Similarly, Idrissi et al. (50) observed analogous results, noting increased triglyceride levels and decreased diacylglycerol concentrations in grade 1 bovine embryos.

## 5 Conclusion

The result of this study demonstrates that high-AFC Sahiwal cows have a larger diameter of preovulatory follicles and greater corpus luteum size. The antral follicle population showed a strong positive correlation with ovarian size, superovulatory response, and embryo production with more transferable embryos. Based on these findings, AFC is concluded to be a reliable phenotypic marker to predict the reproductive potential of Sahiwal donors. This is a crucial factor to take into account in commercial programs aiming for *in vivo* embryo production.

## Data Availability

The original contributions presented in the study are included in the article/supplementary material, further inquiries can be directed to the corresponding author.
